# Density-Dependent Effects of Amphibian Prey on the Growth and Survival of an Endangered Giant Water Bug

**DOI:** 10.3390/insects2040435

**Published:** 2011-09-30

**Authors:** Shin-ya Ohba

**Affiliations:** Center for Ecological Research, Kyoto University, 2-509-3 Hirano, Otsu 520-2113, Japan; E-Mail: oobug@hotmail.com

**Keywords:** aquatic insects, density-dependent indirect effect, intraguild predation, life history, rice fields, temporary water

## Abstract

Amphibian predator-insect prey relationships are common in terrestrial habitats, but amphibian larvae are preyed upon by a variety of aquatic hemipterans in aquatic habitats. This paper suggests that the survival of the nymphs of the endangered aquatic hemipteran *Kirkaldyia (=Lethocerus) deyrolli* (Belostomatidae: Heteroptera) is directly and indirectly affected by the abundance of their amphibian larval prey (tadpoles). Young nymphs of *K. deyrolli* mainly feed on tadpoles, regardless of differences in prey availability. Nymphs provided with tadpoles grow faster than nymphs provided with invertebrate prey. Therefore, tadpole consumption seems to be required to allow the nymphs to complete their larval development. In addition, the survival of *K. deyrolli* nymphs was greater during the period of highest tadpole density (June) than during a period of low tadpole density (July). Higher tadpole density moderates predation pressure from the water scorpion *Laccotrephes japonensis* (Nepidae: Heteroptera) on *K. deyrolli* nymphs; *i.e.*, it has a density-mediated indirect effect. These results suggest that an abundance of tadpoles in June provides food for *K. deyrolli* nymphs (a direct bottom-up effect) and moderates the predation pressure from *L. japonensis* (an indirect bottom-up effect). An abundance of amphibian prey is indispensable for the conservation of this endangered giant water bug species.

## 1. Introduction

### 1.1. Trophic Interactions of Aquatic Insects and Amphibians

The trophic interactions of insects and amphibians have received a significant amount of attention by researchers. Amphibian adults eat a variety of terrestrial arthropods. Insects with a range of gape sizes [1,2] that co-exist in the same habitats as amphibians are exposed to high predation pressure from amphibians [3]. Generally, although amphibians (mainly those of the post-metamorphic stage) are sometimes preyed on by insects ([4], reviewed in Toledo [5]), amphibian predator–insect prey relationships are common in terrestrial habitats. In contrast, these predator–prey relationships are reversed for larval amphibians in aquatic habitats; *i.e.*, amphibian larvae are preyed upon by a variety of aquatic insects such as Coleoptera [6], Heteroptera [7], and Odonata [8]. Amphibian larvae are keystone organisms for aquatic communities because they are important prey resources for aquatic insects. Insect predator-anuran larvae prey relationships in aquatic environments are appropriate model systems for examining predator–prey relationships.

Amphibian larvae show anti-predatory behavior (low activity) and morphological changes (larger heads) in response to predators in order to increase their chances of survival [9-16]. In a study of anurans, Vonesh *et al.* reported that reductions in larval density and size due to egg-stage predators facilitate larval survival in the presence of aquatic predators (predator-induced hatching plasticity) [17-19]. Thus, insect predator-anuran larvae prey relationships have been studied from the viewpoint of behavior and phenotypic plasticity in anuran larvae. However, very few studies have investigated the density-mediated indirect effects of insect predator–anuran larvae prey relationships. Here, I focused on insect predator–anuran larvae prey relationships from the viewpoint of density-mediated indirect effects.

### 1.2. Are There any Aquatic Heteropteran Predators of Amphibian Larvae?

Generally, dragonfly nymphs are mainly used as insect predators of amphibians in model predator–prey systems [9-12]. In addition, aquatic hemipterans play a significant role as the major component of the aquatic fauna of aquatic environments that are devoid of fish [20,21] and are often at the top of the food chain in such aquatic communities, preying upon a variety of aquatic animals [22,23]. As with Odonata predators, some researchers studied phenotypic changes in amphibians induced by aquatic hemipterans predators [24-26]. Certain environments also reduce predation by aquatic hemipteran predators. Kopp *et al.* [28] showed aquatic vegetation reduces predation rates on tadpoles by aquatic hemipteran predators. Swart and Taylor [27] revealed that tadpoles switched their preference away from black backgrounds in response to chemical signals from a predator (aquatic hemipterans) because the predator killed significantly more tadpoles on dark backgrounds than on light backgrounds. Therefore, aquatic hemipteran predators are regarded as important predators for anuran larvae.

**Figure 1:**
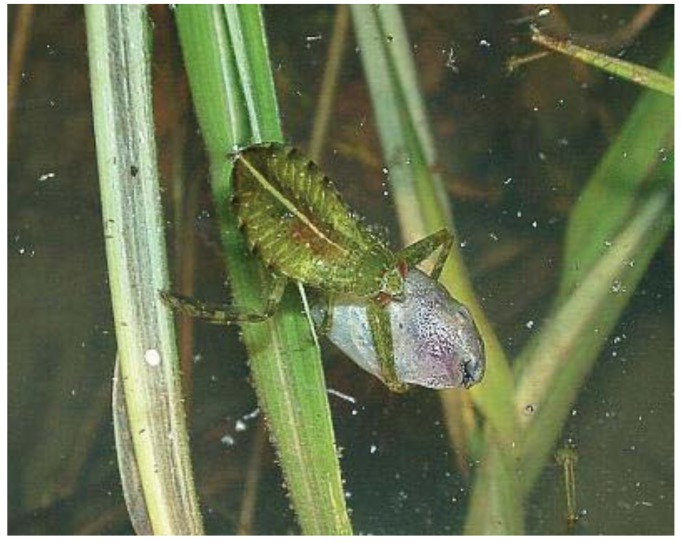
Predation on a tadpole by an aquatic insect. As one example of the recorded dietary items, prey into which a predator’s proboscis had been inserted is shown in this photo.

**Figure 2:**
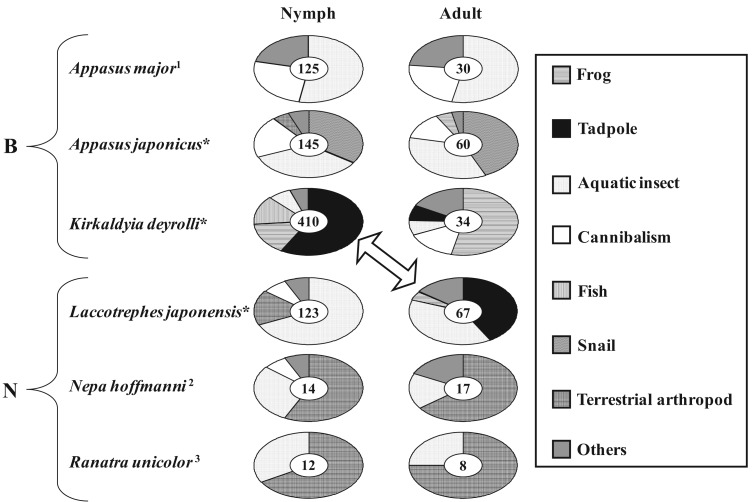
Differences in dietary components among predacious aquatic Heteroptera inhabiting Japanese wetlands (modified from Ohba and Nakasuji [7]). The numbers in the center of each circular graph indicate the sample size. *B*, Belostomatidae; *N*, Nepidae; *1*, Okada and Nakasuji [29]; *2*, Ban *et al.* [31]; *3* Ban [30], * sympatric species. The arrow indicates the relationship between intraguild predators preying upon tadpoles.

However, most aquatic hemipterans do not often eat tadpoles in Japan. Ohba and Nakasuji [7] investigated the feeding habits of aquatic bugs (Nepoidea, including Belostomatidae and Nepidae) by performing direct observations in wetland areas (see Figure 1) and obtaining data from the published literature [29-31]. As a result, it was found that sympatric species (*Appasus japonicus*, *Kirkaldyia deyrolli*, and *Laccotrephes japonensis*) displayed differences in their dietary components (Figure 2). Although tadpoles are preyed upon by a variety of aquatic insects, not all aquatic insect species eat tadpoles. Only *K. deyrolli* nymphs and *L. japonensis* adults greatly depend on tadpoles whereas *A. japonicus* does not eat tadpoles. Therefore, *K. deyrolli* nymphs and *L. japonensis* adults seem to be members of the same guild; *i.e.*, they compete with each other, in Japanese wetlands [32].

In this paper, I first introduce that *K. deyrolli* nymphs feed on tadpoles. Second, I introduce that *K. deyrolli* nymph survival is indirectly affected by tadpoles; *i.e.*, by a density-mediated indirect effect. Finally, I discuss aquatic heteropteran predator–amphibian larval prey relationships in relation to temporal dynamic interactions.

## 2. Tadpole-Feeding by *K. deyrolli* Nymphs

The quantity and quality of prey animals strongly affect a predator’s life history and abundance. Among predacious insects, predatory species that depend upon a particular prey animal, which are known as specialists, breed when their prey animal is abundant [33-36]. Specialist development coincides with the appearance of specific prey animals, especially during the nymphal period [37]. In rice fields, *K. deyrolli* nymphs prey upon tadpoles more than on other kinds of prey (Figure 2). The subfamily Lethocerinae, which has the largest body size among Belostomatidae, is known to be a vertebrate specialist [38-40]. Why *K. deyrolli* nymphs mostly feed on tadpoles is not well understood from the viewpoint of nymphal growth and seasonal occurrence.

**Figure 3:**
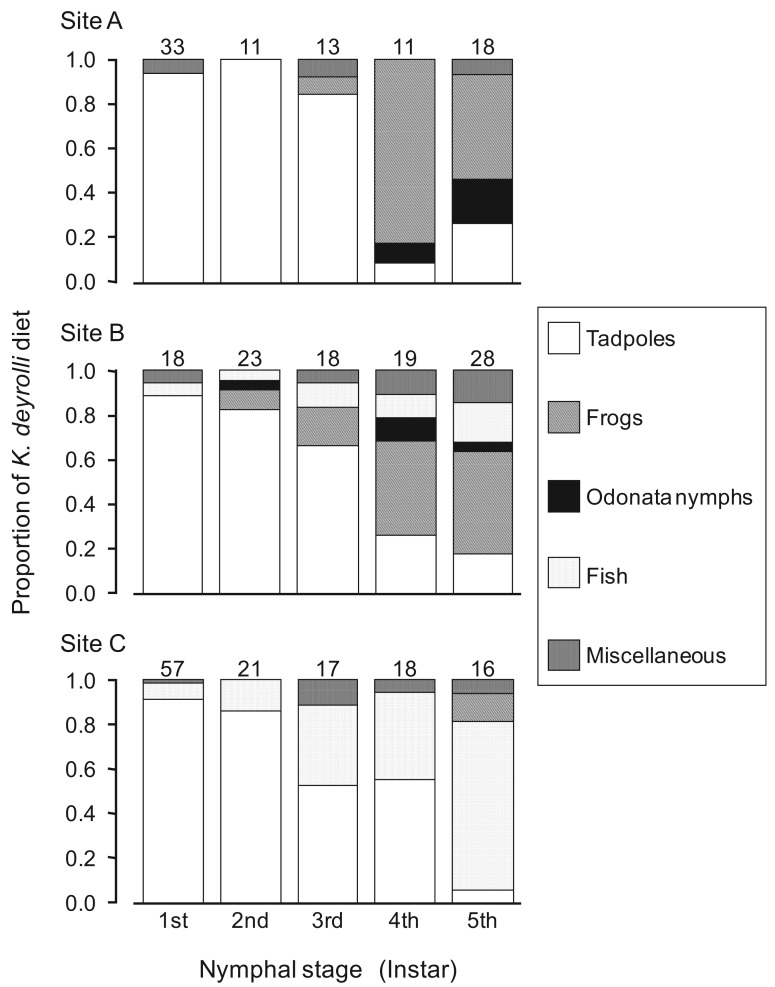
Prey groups included in the diets of *K. deyrolli* nymphs at three localities (modified from Ohba *et al.* [45]). The numbers indicate sample sizes.

**Figure 4:**
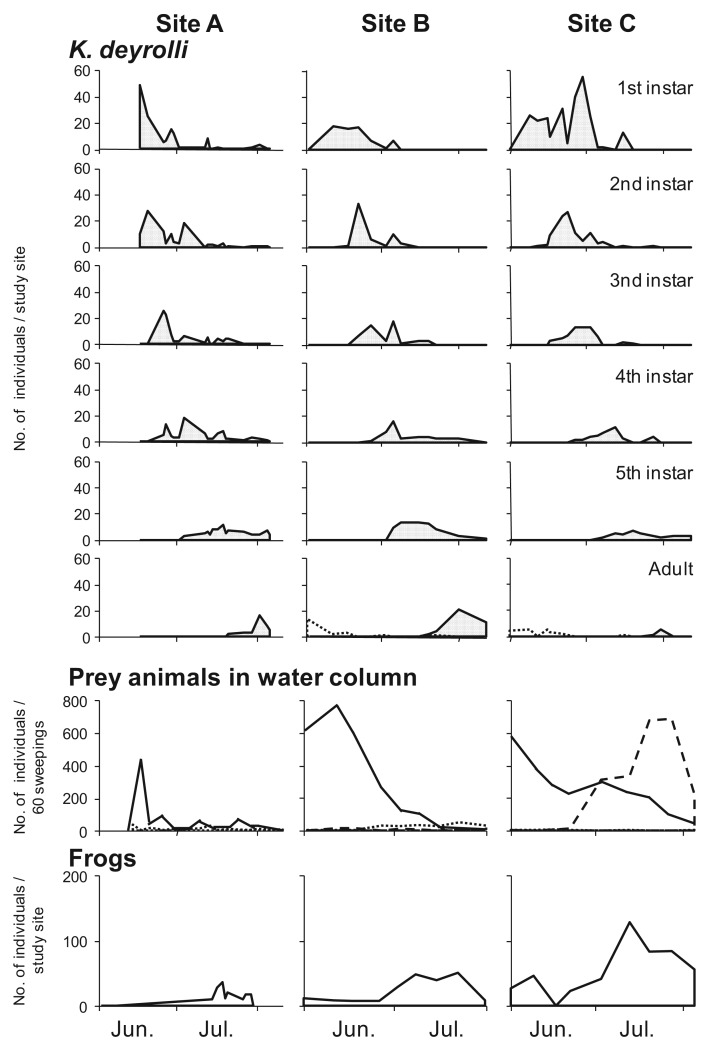
Seasonal changes in the frequencies of *K. deyrolli* and four prey categories at the three study sites. For the adult *K. deyrolli* graphs, the solid lines indicate overwintered insects, and the broken lines denote newly emerged adults. In the prey animals in the water column graphs, the solid, broken, and dotted lines indicate tadpoles, fish, and Odonata nymphs, respectively (modified from Ohba *et al.* [45]).

In general, the appearance of younger nymphs of predacious insects when prey animals are abundant is expected to moderate cannibalism due to food shortages [41-44]. Accordingly, Ohba *et al.* [45] studied the ontogenetic diet shift of *K. deyrolli* by quantifying instar abundance and analyzing captured prey and prey relative abundance in rice fields in three localities. The first to third-instar *K. deyrolli* nymphs mainly fed on tadpoles, regardless of differences in prey availability among the three localities (Figure 3). A rearing experiment demonstrated that *K. deyrolli* nymphs provided with tadpoles displayed greater growth rates at all nymphal stages, except for the final stage, than nymphs fed on dragonflynymphs. The emergence of young *K. deyrolli* nymphs seemed to coincide with the period when tadpoles became abundant in the rice fields (Figure 4). In addition, the appearance of younger *K. deyrolli* nymphs when tadpoles are abundant is expected to moderate cannibalism due to food shortages, as has been demonstrated for other predatory insects such as ladybirds [41-44]. Actually, the frequency of cannibalism in *K. deyrolli* nymphs is lower than that seen in *A. japonicus* nymphs in the field (Figure 2) [7].

## 3. Density-Mediated Indirect Effects of Tadpole Prey

Many specialists breed when certain prey are abundant in order to increase the chance of there being a sufficient amount of food to increase the growth and survival of their young, showing that a high prey density directly supports the predator population, e.g., a bottom-up effect. However, there is little information on the indirect role of higher prey density on the mediation of predation pressure on the predator from other predators within the same guild; *i.e.*, density-mediated indirect effects, in aquatic environments including insect predator–anuran larva prey relationships. As mentioned above, *K. deyrolli* nymphs are considered to feed on tadpoles [45]. In addition, *L. japonensis* prey upon *K. deyrolli* nymphs [32,46] and tadpoles in rice fields [7]. Therefore, *L. japonensis* adults are intraguild predators of *K. deyrolli* nymphs. Consequently, it is predicted that the survivorship of *K. deyrolli* nymphs is affected by their common prey, tadpoles, via a bottom-up effect on survivorship as well as by their predator, *L. japonensis* adults, via a top-down effect. Thus, it is considered that density-mediated indirect relationships exist among the three animal species.

Ohba and Nakasuji [47] demonstrated that the survival of *K. deyrolli* nymphs, which are tadpole specialists, is affected by tadpoles (a direct bottom-up effect). We investigated the survival rates of the first instar nymphs of *K. deyrolli* in June (high tadpole density period) and July (low tadpole density period) using the Kiritani-Nakasuji-Manly method [48,49]. As circumstantial evidence, the survival rate of the first instar nymphs of *K. deyrolli* in a rice paddy field was higher in June (75.1%) than in July (54.2%) [47]. The differences in prey density between June and July probably affected the survival rates of the *K. deyrolli* nymphs, suggesting that bottom-up effects display seasonal variation.

**Figure 5:**
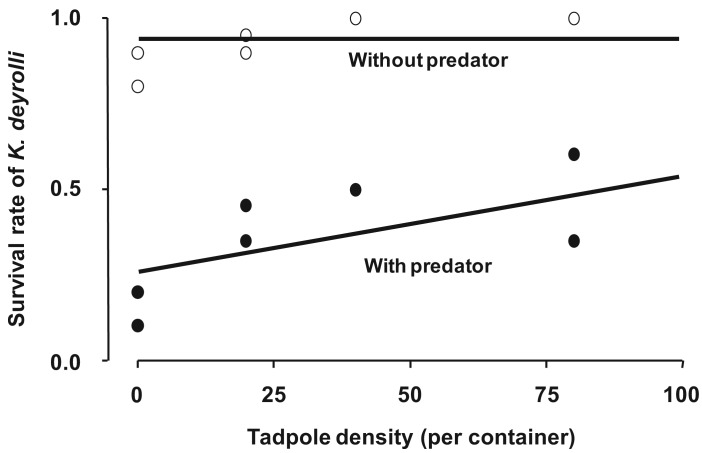
Effects of tadpole density and the presence or absence of *L. japonensis* adults (a *K. deyrolli* nymph predator) on *K. deyrolli* nymph survival rates. The regression lines were calculated using a logistic regression model. (modified from Ohba and Nakasuji [47]).

**Figure 6:**
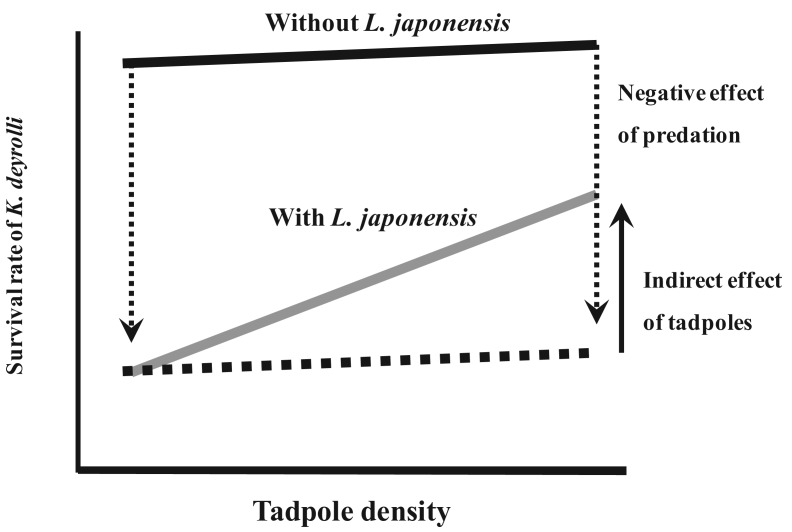
Schematic representation of the indirect effects of tadpole density on the survivorship of *K. deyrolli* nymphs in the presence or absence of their predator *L. japonensis* adults (modified from Ohba and Nakasuji [47]).

To examine whether a higher tadpole density moderates the predation pressure from *L. japonensis* adults on *K. deyrolli* nymphs, a field experiment was conducted in a rice field. As a result, it was found that a higher tadpole density moderated the predation pressure from *L. japonensis* adults on *K. deyrolli* nymphs (a density-mediated indirect effect; Figures 5 and 6) [50,51]. Prey abundance is an important factor for determining the frequency of intraguild predation [52-54], especially in terrestrial aphidophagous predator–aphid prey relationships. Thus, in rice fields the incidence of intraguild predation by *L. japonensis* adults might increase when the tadpole density decreases.

## 4. Conservation of an Endangered Giant Water Bug

The Japanese populations of *K. deyrolli* have decreased sharply during the last four decades, and this species is now included in the *Red Data List* of species in 45 of 47 Japanese prefectures [55,56]. Contributing factors such as decreases in suitable aquatic habitats, water pollution, and urbanization have been investigated and verified in previous studies [57-59]. In addition to these factors, it is important for the conservation of *K. deyrolli* to reveal the best food for them to eat. Regarding bottom-up effects, Hirai and Hidaka [60] and Hirai [61] emphasized that the frog population is very important for the conservation of the *K. deyrolli* population because frogs are major constituents of the diet of *K. deyrolli* adults.

Previous studies have only focused on direct bottom-up effects of amphibian prey. However, the present study demonstrated an indirect bottom-up effect of amphibian prey. The emergence of young *K. deyrolli* nymphs appeared to coincide with the period when tadpoles became abundant in the rice fields, which allowed the young *K. deyrolli* nymphs to achieve greater growth (Figure 4 [45]). This indicates that a high prey density directly supports *K. deyrolli* nymphs. In addition to such direct effects, a high tadpole density was also demonstrated to have a beneficial indirect effect on the survival of *K. deyrolli* nymphs (Figure 6 [47]). Therefore, an abundance of amphibian prey is indispensable for the conservation of this endangered giant water bug species.

## 5. Conclusions

This paper introduced that young *K. deyrolli* nymphs mainly feed on tadpoles in rice fields and that nymphs provided with tadpoles grow faster than nymphs provided with invertebrate prey. A higher tadpole density moderates the predation pressure from the water scorpion *L. japonensis* on *K. deyrolli* nymphs; *i.e.*, it has a density-mediated indirect effect. These results suggest that an abundance of tadpoles provides sufficient food for *K. deyrolli* nymph growth (a direct bottom-up effect) and moderates the predation pressure exerted on them by *L. japonensis* (an indirect bottom-up effect). An abundance of amphibian prey is indispensable for the conservation of this endangered giant water bug species.

## References

[1] Toft C.A. (1980). Feeding ecology of thirteen syntopic species of anurans in a seasonal tropical environment. Oecologia.

[2] Hirai T. (2002). Ontogenetic change in the diet of the pond frog, *Rana nigromaculata*. Ecol. Res..

[3] Honma A., Oku S., Nishida T. (2006). Adaptive significance of death feigning posture as a specialized inducible defence against gape-limited predators. Proc. R. Soc. Biol. Sci. Ser. B.

[4] Benard M. (2007). Predators and mates: Conflicting selection on the size of male Pacific treefrogs (*Pseudacris regilla*). J. Herpetol..

[5] Toledo L. (2003). Predation on seven South American anuran species by water bugs (Belostomatidae). Phyllomedusa.

[6] Ohba S. (2009). Ontogenetic dietary shift in the larvae of *Cybister japonicus* (Coleoptera: Dytiscidae) in Japanese rice fields. Environ. Entomol..

[7] Ohba S., Nakasuji F. (2006). Dietary items of predacious aquatic bugs (Nepoidea: Heteroptera) in Japanese wetlands. Limnology.

[8] Corbet P.S. (1999). Dragonflies: Behaviour and Ecology of ODONATA.

[9] van Buskirk J., McCollum S. (1999). Plasticity and selection explain variation in tadpole phenotype between ponds with different predator composition. Oikos.

[10] Van Buskirk J., Relyea R. (1998). Selection for phenotypic plasticity in *Rana sylvatica* tadpoles. Biol. J. Linn. Soc..

[11] van Buskirk J. (2002). Phenotypic lability and the evolution of predator-induced plasticity in tadpoles. Evolution.

[12] McIntyre P., Baldwin S., Flecker A. (2004). Effects of behavioral and morphological plasticity on risk of predation in a Neotropical tadpole. Oecologia.

[13] Kishida O., Nishimura K. (2004). Bulgy tadpoles: Inducible defense morph. Oecologia.

[14] Takahara T., Kohmatsu Y., Maruyama A., Yamaoka R. (2008). Benefit of suites of defensive behavior induced by predator chemical cues on anuran tadpoles. Hyla Jpn. Behav. Ecol. Sociobiol..

[15] Hettyey A., Zsarnoczai S., Vincze K., Hoi H., Laurila A. (2010). Interactions between the information content of different chemical cues affect induced defences in tadpoles. Oikos.

[16] Jara F.G., Perotti M.G. (2009). Toad tadpole responses to predator risk: Ontogenetic change between constitutive and inducible defenses. J. Herpetol..

[17] Vonesh J.R. (2005). Egg predation and predator-induced hatching plasticity in the African reed frog, *Hyperolius spinigularis*. Oikos.

[18] Vonesh J.R. (2005). Sequential predator effects across three life stages of the African tree frog, *Hyperolius spinigularis*. Oecologia.

[19] Vonesh J.R., Bolker B.M. (2005). Compensatory larval responses shift trade-offs associated with predator-induced hatching plasticity. Ecology.

[20] Runck C., Blinn D.W. (1994). Role of *Belostoma bakeri* (Heteroptera) in the trophic ecology of a fishless desert spring. Limnol. Oceanogr..

[21] Blaustein L. (1998). Influence of the predatory backswimmer, *Notonecta maculata*, on invertebrate community structure. Ecol. Entomol..

[22] Waters T.F. (1977). Secondary production in inland waters 1. Adv. Ecol. Res..

[23] Runck C., Blinn W. (1990). Population dynamics and secondary production by *Ranatra montezuma* (Heteroptera: Nepidae). J. North. Am. Benthol. Soc..

[24] Gomez V.I., Kehr A.I. (2011). Morphological and developmental responses of anuran larvae (*Physalaemus albonotatus*) to chemical cues from the predators *Moenkhausia dichoroura* (Characiformes: Characidae) and *Belostoma elongatum* (Hemiptera: Belostomatidae). Zool. Stud..

[25] Kehr A.I., Gómez V.I. (2009). Intestinal, body and tail plasticity in *Rhinella schneideri* (Bufonidae) tadpoles induced by a predator insect (*Belostoma elegans*). Adv. Stud. Biol..

[26] Hoverman J.T., Relyea R.A. (2008). Temporal environmental variation and phenotypic plasticity: A mechanism underlying priority effects. Oikos.

[27] Swart C.C., Taylor R.C. (2004). Behavioral interactions between the giant water bug (*Belostoma lutarium*) and tadpoles of *Bufo woodhousii*. Southeast. Nat..

[28] Kopp K., Wachlevski M., Eterovick P.C. (2006). Environmental complexity reduces tadpole predation by water bugs. Can. J. Zool..

[29] Okada H., Nakasuji F. (1993). Comparative studies on the seasonal occurrence, nymphal development and food menu in two giant water bugs, *Diplonychus japonicus* Vuillefroy and *Diplonychus major* Esaki (Hemiptera: Belostomatidae). Res. Popul. Ecol..

[30] Ban Y. (1981). Some observation on the life cycle of the water scorpion, *Ranatra unicolor* Scott (Hemiptera: Nepidae). Yamanoshita Bay, Lake Biwa. Verh. Int. Verein. Limnol..

[31] Ban Y. (1988). Water Scorpion NEPA Hoffmani.

[32] Ohba S. (2007). Notes on predators and their effect on the survivorship of the endangered giant water bug, *Kirkaldyia (=Lethocerus) deyrolli* (Heteroptera, Belostomatidae), in Japanese rice fields. Hydrobiologia.

[33] Evans E. (1982). Feeding specialization in predatory insects: Hunting and attack behavior of two stinkbug species (Hemiptera: Pentatomidae). Am. Midl. Nat..

[34] Sota T. (1985). Activity patterns, diets and interspecific interactions of coexisting spring and autumn breeding carabids: *Carabus yaconinu*s and *Leptocarabus kumagaii* (Coleoptera, Carabidae). Ecol. Entomol..

[35] Hagen K.S. (1962). Biology and ecology of predaceous Coccinellidae. Annu. Rev. Entomol..

[36] Albuquerque G., Tauber M., Tauber C. (1997). Life-history adaptations and reproductive costs associated with specialization in predacious insects. J. Anim. Ecol..

[37] Elkinton J.S., Resh V.H., Carde R.T. (2003). Population Ecology. Encyclopedia of Insects.

[38] Smith R.L., Choe J., Crespi B. (1997). Evolution of Paternal Care in the Giant Water Bugs (Heteroptera: Belostomatidae). The evolution of social behavior in insects and arachnids.

[39] Swart C.C., Deaton L.E., Felgenhauer B.E. (2006). The salivary gland and salivary enzymes of the giant waterbugs (Heteroptera; Belostomatidae). Comp. Biochem. Physiol. Part A Mol. Integr. Physiol..

[40] Ohba S. (2011). Field observation of predation on a turtle by a giant water bug. Entomol. Sci..

[41] Takahashi K. (1989). Intra- and inter-specific predations of lady beetles in spring alfalfa fields. Jpn. J. Entomol..

[42] Osawa N. (1992). A life table of the ladybird beetle *Harmonia axyridis* Pallas (Coleoptera, Coccinellidae) in relation to the aphid abundance. Jpn. J. Entomol..

[43] Agarwala B., Dixon A. (1992). Laboratory study of cannibalism and interspecific predation in ladybirds. Ecol. Entomol..

[44] Hironori Y., Katsuhiro S. (1997). Cannibalism and interspecific predation in two predatory ladybirds in relation to prey abundance in the field. Entomophaga.

[45] Ohba S., Miyasaka H., Nakasuji F. (2008). The role of amphibian prey in the diet and growth of giant water bug nymphs in Japanese rice fields. Popul. Ecol..

[46] Ohba S., Swart C.C. (2009). Intraguild predation of water scorpion *Laccotrephes japonensis* (Nepidae: Heteroptera). Ecol. Res..

[47] Ohba S., Nakasuji F. (2007). Density-mediated indirect effects of a common prey tadpole on interaction between two predatory bugs: *Kirkaldyia deyrolli* and *Laccotrephes japonensis*. Popul. Ecol..

[48] Kiritani K., Nakasuji F. (1967). Estimation of the stage-specific survival rate in the insect population with overlapping stages. Res. Popul. Ecol..

[49] Manly B.F.J. (1976). Extensions to Kiritani and Nakasuji's method for analysing insect stage-frequency data. Res. Popul. Ecol..

[50] Holt R., Lawton J. (1994). The ecological consequences of shared natural enemies. Annu. Rev. Ecol. Syst..

[51] Holt R.D. (1977). Predation, apparent competition, and the structure of prey communities. Theor. Popul. Biol..

[52] Polis G., Myers C., Holt R. (1989). The ecology and evolution of intraguild predation: Potential competitors that eat each other. Annu. Rev. Ecol. Syst..

[53] Lucas E., Coderre D., Brodeur J. (1998). Intraguild predation among aphid predators: Characterization and influence of extraguild prey density. Ecology.

[54] Hodge M.A. (1999). The implications of intraguild predation for the role of spiders in biological control. J. Arachnol..

[55] Search system of Japanese red data. http://www.jpnrdb.com/index.html.

[56] Japan Environment Agency (2000). Threatened Wildlife of Japan. Red Data Book.

[57] Ho C., Kim H., Kim J.G. (2009). Landscape analysis of the effects of artificial lighting around wetland habitats on the giant water bug *Lethocerus deyrollei* in Jeju Island. J. Ecol. Field. Biol..

[58] Yoon T., Kim D., Kim S., Jo S., Bae Y. (2010). Light-attraction flight of the giant water bug, *Lethocerus deyrolli* (Hemiptera: Belostomatidae), an endangered wetland insect in east Asia. Aquat. Insects.

[59] Nagaba Y., Tufail M., Inui H., Takeda M. (2011). Hormonal regulation and effects of four environmental pollutants on vitellogenin gene transcription in the giant water bug, *Lethocerus deyrollei* (Hemiptera: Belostomatidae). J. Insect Conserv..

[60] Hirai T., Hidaka K. (2002). Anuran-dependent predation by the giant water bug, *Lethocerus deyrollei* (Hemiptera: Belostomatidae), in rice fields of Japan. Ecol. Res..

[61] Hirai T. (2007). Diet composition of the endangered giant water bug *Lethocerus deyrolli* (Hemiptera: Belostomatidae) in the rice fields of Japan: Which is the most important prey item among frogs, fish, and aquatic insects?. Entomol. Sci..

